# Systematic Review and Meta-Analysis on the Frequency of Antibiotic-Resistant *Clostridium* Species in Saudi Arabia

**DOI:** 10.3390/antibiotics11091165

**Published:** 2022-08-29

**Authors:** Saeed S. Banawas

**Affiliations:** 1 Department of Medical Laboratories, College of Applied Medical Science, Majmaah University, Al-Majmaah 11952, Saudi Arabia; s.banawas@mu.edu.sa; Tel.: +966-164041510; 2 Health and Basic Sciences Research Center, Majmaah University, Al-Majmaah 11952, Saudi Arabia; 3 Department of Biomedical Sciences, Oregon State University, Corvallis, OR 97331, USA

**Keywords:** antibiotic resistance, Saudi Arabia, *Clostridium difficile*, *Clostridium perfringens*, *Clostridium tetani*, *Clostridium botulinum*

## Abstract

*Clostridium* is a genus comprising Gram-positive, rod-shaped, spore-forming, anaerobic bacteria that cause a variety of diseases. However, there is a shortage of information regarding antibiotic resistance in the genus in Saudi Arabia. This comprehensive analysis of research results published up until December 2021 intends to highlight the incidence of antibiotic resistance in *Clostridium* species in Saudi Arabia. PubMed, Google Scholar, Web of Science, SDL, and ScienceDirect databases were searched using specific keywords, and ten publications on antibiotic resistance in *Clostridium* species in Saudi Arabia were identified. We found that the rates of resistance of *Clostridium difficile* to antibiotics were as follows: 42% for ciprofloxacin, 83% for gentamicin, 28% for clindamycin, 25% for penicillin, 100% for levofloxacin, 24% for tetracycline, 77% for nalidixic acid, 50% for erythromycin, 72% for ampicillin, and 28% for moxifloxacin; whereas those of *C. perfringens* were: 21% for metronidazole, 83% for ceftiofur, 39% for clindamycin, 59% for penicillin, 62% for erythromycin, 47% for oxytetracycline, and 47% for lincomycin. The current findings suggest that ceftiofur, erythromycin, lincomycin, and oxytetracycline should not be used in *C. perfringens* infection treatments in humans or animals in Saudi Arabia.

## 1. Introduction

*Clostridium* is a genus of Gram-positive, rod-shaped, anaerobic bacteria that cause a wide variety of diseases in humans and animals [1,2]. Furthermore, antibiotic-resistant bacteria are considered one of the biggest threats to global health, food safety, and development [3]. Bacteria are unicellular microorganisms that can develop resistance to antibacterial agents; unfortunately, the misuse of these antibacterials in both humans and animals is accelerating this process [2]. Several factors play key roles in the emergence of resistance. One of them is the acquisition of new genes from other bacterial strains, which helps a bacterium develop new mechanisms to withstand/counteract antibacterial actions against it [4]. Another factor is the alteration of bacterial DNA due to random mutations, which enables bacteria to survive, better withstand a variety of environments, and multiply [4].

Antibacterial-resistant strains of *Clostridium,* including *C. difficile*, *C. tetani, C. perfringens,* and *C. botulinum*, have been reported worldwide [2]. *C. difficile* is a strictly anaerobic pathogenic bacterium that produces spores that can survive in the environment for years; it is highly resistant and causes *C. difficile* infections (CDIs) [5]. *C. difficile* is an important antimicrobial-resistant microorganism associated with watery diarrhea and pseudomembranous colitis infections in healthcare facilities, causing mild to moderate and sometimes life-threatening disease [6,7,8]. It is part of the intestinal microbiota of approximately 3% of healthy adults and 20% of infants [9]. CDI is the major cause of approximately 33% of cases of antibiotic-associated diarrhea (AAD) and 90% of cases of pseudomembranous enteritis [10]. Disturbance of the gut microbiota caused by broad-spectrum antibiotics is thought to originate in 33% of AADs, allowing *C. difficile* to colonize and grow in the colon, resulting in disease symptoms.

*C. difficile* is the major bacterial cause of hospital-acquired diarrheas associated with antibiotics, not only in the United States, but worldwide [2,11]. The Centers for Disease Control and Prevention (CDC) and other organizations have reported that, yearly, antibiotic-resistant CDIs occur in approximately 2.8 million patients and lead to more than 35,000 deaths in the United States [11,12]. The annual CDI-attributable cost to the global healthcare system is more than USD 4.6 billion [11,12]. Despite the existence of clear guidelines as well as diagnostic and therapeutic approaches, the CDI rate is increasing, both in Europe and the United States [13]. The clinical signs and symptoms of CDI range from mild to severe diarrhea, which may lead to toxic megacolon, sepsis, fulminant colitis, bowel perforation, and even death [14].

Although no nationwide investigation on CDI prevalence has been published in Saudi Arabia, the few single-center studies that have been published have revealed a low rate of CDIs [15,16,17]. Of all diarrhea samples evaluated, one of these studies was able to find a rising trend of healthcare-associated CDIs, from 17% in 2001 to 20% in 2018 [16]. Reports suggest the emergence of *C. difficile* multidrug-resistant strains with high morbidity and mortality rates. One such strain is the NAP1/BI/027 strain (also known as ribotype [RT] 027), which was first reported in England in 2005 [18,19]; since then, it has rapidly spread to other European countries and the USA. This strain has also been reported in Saudi Arabia [20], where it affected four elderly patients, one of whom was administered metronidazole and vancomycin; however, the patient’s condition worsened, and he eventually died. The erythromycin and moxifloxacin resistance exhibited by the RT 027 strain may confer a selective advantage for the bacterium [21]. RT 027 resistance to metronidazole, moxifloxacin, and vancomycin has been reported in several countries [22,23,24,25,26,27,28,29].

According to recent statistics, strain RT 027 is the most prevalent hypervirulent strain, causing serious infections and increasing mortality globally [30,31,32,33]. Another highly virulent strain, RT 078, which causes infections in humans (especially in hospitals and communities) and animals, was found in European countries such as the Netherlands [34,35,36,37,38]. The spread of CDIs among hospitalized patients and the elderly has been attributed to antibiotic resistance. Antibiotic resistance also affects healthy individuals [39]. In recent years, different countries have reported an increase in CDI incidence among their residents. In addition, domestic clinical isolates of the RT 017 strain have been commonly observed in the USA, Canada, Poland, and South Korea [24,25,40,41,42].

Treatment with broad-spectrum antimicrobial medicines, which produce an imbalance in the intestinal microflora, is the most common risk-enhancing factor for CDI due to developed resistance. Antimicrobial drug medications, such as erythromycin, penicillin, cephalosporins, clindamycin, and fluoroquinolones, stimulate an extensive CDI transmission inside and outside of hospitals and enhance *C. difficile* resistance to these drugs [43,44,45,46]. *C. difficile* resistance to erythromycin, penicillin, cephalosporins, clindamycin, and fluoroquinolones has already been reported in some studies [47,48,49]. For example, in New Zealand, approximately 100 isolates collected from 97 patients showed 100% resistance to penicillin [49]. Another study in Sweden showed that approximately 85% of the isolates that were collected from 13 patients with *C. difficile*-associated diarrhea were resistant to clindamycin [47]. One study reported that *C. difficile* isolates from different countries showed different tetracycline-resistance profiles, which ranged from 0 to 39% of the strains being resistant [50,51].

Most individuals with CDI are treated with metronidazole or vancomycin to ensure the best treatment outcome. However, recurrent use of these drugs will sooner or later result in the development of *C. difficile* resistant strains. Currently, metronidazole and fidaxomicin are the most widely used drugs to treat patients with CDIs, whereas the use of vancomycin is avoided [11]. Though the use of vancomycin usually decreases the infection in more than 95% of the cases, it leads to CDI recurrence in 15–30% of individuals [52,53,54,55]. This is because the high selectivity of vancomycin-resistant *Enterococcus* [56,57] may also lead to the formation of *C. difficile* vancomycin-resistant strains (*e.g*., by horizontal transfer).

Studies from different countries have demonstrated an increase in *C. difficile* resistance to vancomycin. In Poland, approximately 8% of patients with CDI were found to have strains resistant to vancomycin (three out of 38 isolates) [58]. In Spain, approximately 6% of CDI sample isolates showed intermediate resistance to vancomycin [56]. In addition, vancomycin resistance was observed in approximately 58 and 31.5% of strains in Brazil and Israel, respectively. [59,60]. In Iran, the percentage of *C. difficile* strains isolated from human samples that were resistant to vancomycin increased from 8 to 20% from 2010 to 2016 [61,62,63].

*C. perfringens* is a species associated with foodborne illnesses, gas gangrene, and various other diseases in both humans and animals [1,2,64,65,66,67]. This bacterium is usually found in meat products, and its spores can survive cooking processes and thus germinate and multiply rapidly [68,69,70,71]. In humans, soft tissue wound infections, such as cellulitis, suppurative myositis, and myonecrosis, are caused by *C. perfringens* [1,2,65,66]. In addition, *C. perfringens* affects livestock, causing diseases such as necrotic enteritis, and has an impact at the economic level [72].

*C. perfringens* isolates are classified into seven different toxinotypes (named A–G) depending on the major types of toxins they produce (alpha, beta, epsilon, or iota). Overall, *C. perfringens* strains can secrete more than 20 extracellular toxins or hydrolytic enzymes, which are the main virulence factors involved in their pathogenicity [66,73,74,75]. Type F isolates, for example, produce *C. perfringens* enterotoxin (CPE), which is responsible for food poisoning (FP) and non-foodborne (NFB) gastrointestinal (GI) diseases [1,76,77,78,79]. Approximately 5% to 15% of AAD cases are linked to *C. perfringens* type F FP and NFB GI diseases [1,76,77,78,79,80,81]. Interestingly, most cases of FP are caused by type F isolates carrying the CPE-encoding gene (*cpe*) on chromosome C (C-*cpe* isolates), whereas NFB GI diseases (i.e., sporadic diarrhea and AAD) are caused by type F isolates carrying a plasmid-borne *cpe* (P-*cpe* isolates) [69,82,83,84]. *C. perfringens* type F FP is the second most commonly reported bacterial foodborne disease and accounts for more than 1 million cases per year in the United States [85,86,87].

Several studies have determined the antimicrobial resistance of *C. perfringens* and the risks that this resistance poses to humans and animals [88]. Resistance to common antibiotics such as penicillin has been frequently reported. Furthermore, resistance to alternative medicines, such as metronidazole and clindamycin, has been observed in isolates from human fecal samples [89,90,91]. In Thailand, most *C. perfringens* isolates from pig and human feces were resistant to tetracycline (approximately 77% and 45%, respectively) [92]. In Egypt, a study on broiler chicken samples revealed that almost 100% of the sample isolates were resistant to gentamicin, oxolinic acid, streptomycin, erythromycin, lincomycin, and spiramycin [93]. A similar study reported that the rates of resistance to lincomycin and tetracycline of *C. perfringens* isolates from broiler chickens were 63% and 66%, respectively [94]. In India, a study reported that more than 55% and 27% of the total *C. perfringens* isolates from ducks were resistant to penicillin and tetracycline, respectively [95].

*C. botulinum* is the causative agent of botulism, a rare but serious paralytic disease (flaccid paralysis) that can affect both humans and animals [96,97,98]. There are three common forms of botulism caused by *C. botulinum.* The first is foodborne botulism, which is caused by bacteria that thrive and produce toxins in an environment where there is little or no oxygen, as in canned foods [96,98]. The second, wound botulism, results from bacteria that enter the body through a wound, where they secrete their toxins, causing a serious wound infection [96]. Finally, infant botulism, the most common form of botulism, starts after *C. botulinum* spores germinate in the intestinal tract of a baby. It typically occurs in babies aged between two and eight months [97,98]. *C. botulinum* toxins are among the most dangerous bacterial toxins that cause paralytic disease [96,99]. According to the latest updated CDC reports, an average of 141 cases of botulism occur in the United States per year, of which approximately 10% are cases of foodborne botulism, 77% are cases of infant botulism, and 10% are cases of wound botulism [100]. *C. botulinum* produces seven antigenically distinguishable exotoxins (A–G) [96,99] that interfere with neural transmission by obstructing the release of acetylcholine, the major neurotransmitter at the neuromuscular junction, resulting in muscle paralysis [96,99]. Recent increases in the number of drug-resistant pathogens have provoked the appearance of highly resistant *C. botulinum* strains, the control of which is challenging [101]. In general, *C. botulinum* strains have been observed to be moderately resistant to chloramphenicol, tetracycline, cephalosporins, and nalidixic acid and highly resistant to cycloserine, nitroimidazoles, gentamicin, sulfamethoxazole, and trimethoprim [102,103].

*C. tetani* is an obligate bacterium that produces two different toxins: tetanolysin, which destroys local tissue, and tetanospasmin, which causes tetanus [104,105,106]. Tetanus is a dangerous disease that affects the nervous system, causing painful muscle contractions, particularly in the jaw and neck muscles [106]. It commonly occurs in geriatric patients but also in young adults in developing countries [104]. Tetanus is now considered rare in developed countries because of vaccination efforts; however, it remains a potentially fatal disease, especially in developing countries [105]. Tetanus is estimated to cause 213,000–293,000 deaths globally each year according to the European Centre for Disease Prevention and Control’s annual epidemiological report, although the number of cases has decreased sharply since the 1950s [107]. *C. tetani* spores are commonly present in the soil and innate objects such as clothing; they are highly resistant and can survive for years. When they enter the body, they activate and produce the potent toxin tetanospasmin, which impairs the nerves that control the muscles and may eventually lead to death [108]. A study showed that approximately 100% of isolates of *C. tetani* from patients were resistant to erythromycin and ofloxacin [109].

In developing countries, including Saudi Arabia, research on the prevalence of antibiotic resistance that could highlight the magnitude of the occurrence of pathogenic *Clostridium* species in humans and animals is lacking. Moreover, antibiotic resistance in pathogenic and commensal bacteria is becoming a serious problem. Therefore, the goal of this research was to determine the prevalence of antibiotic-resistant *Clostridium* species in Saudi Arabia.

## 2. Results

### 2.1. Literature Search

**Figure 1:**
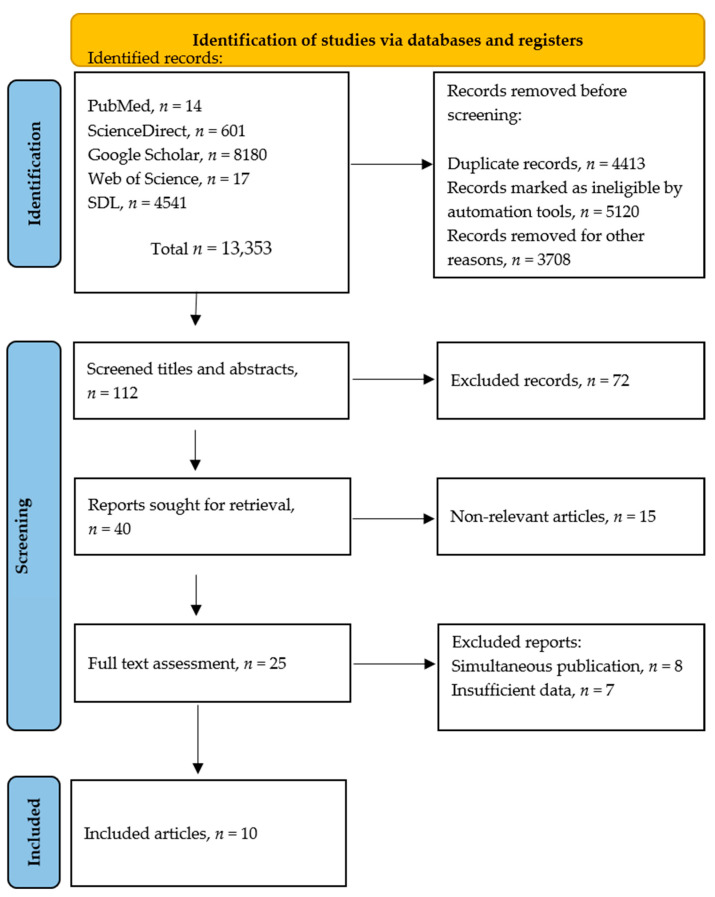
Schematic plan of the article selection process.

As illustrated in Figure 1, an online search was conducted on Google Scholar, PubMed, ScienceDirect, Web of Science, and the SDL databases to find articles on antibiotic resistance in the four Clostridium spp. reported for Saudi Arabia (see Table 1). A total of 13,353 articles were selected after checking their titles and abstracts and applying the inclusion criteria. Of these, only 112 were screened due to their relevance, and 25 of them were further assessed and examined carefully. Finally, we selected 10 studies that were useful for our systematic review and meta-analysis (Figure 1 and Table 2). Any study that lacked crucial data had non-relevant content, or was not local was excluded. Studies on the antibiotic susceptibility of Saudi Arabian *Clostridium* spp., other than *C. difficile* and *C. perfringens*, have not yet been published. 

### 2.2. Characteristics of the Study

Table 2 summarizes the features of the ten selected publications. Al-Ahasa, Jazan, the Eastern Province, Riyadh, Al-Taif, Al-Jouf, and Al-baha were among the cities or regions in which the research was conducted. *C. difficile* and *C. perfringens* were the most common species isolated from animals, humans, stool samples, blood samples, bodily fluids, female genitals, baskets, conveyor belts, and plastic bags. However, we were unable to find any studies on the antibiotic resistance patterns of other Clostridium species in Saudi Arabia. We found that E-tests and disk diffusion assays were the most commonly used methods for antibiotic susceptibility testing, with moxifloxacin, clindamycin, ciprofloxacin, levofloxacin, and erythromycin being the most commonly used antibiotics in these tests.

### 2.3. Antibiotic Resistance Frequencies in C. difficile

In Saudi Arabia, seven studies reported *C. difficile* antibiotic resistance (Table 2). Overall, *C. difficile* strains showed the following average antibiotic resistance percentages: 42% for ciprofloxacin, 83% for gentamicin, 28% for clindamycin, 25% for penicillin, 100% for levofloxacin, 24% for tetracycline, 77% for nalidixic acid, 50% for erythromycin, 72% for ampicillin, and 28% for moxifloxacin. Table 2 shows that the average resistance of *C. difficile* isolates to levofloxacin, gentamicin, erythromycin, and moxifloxacin increased from 2011 to 2020, although the data are limited.

### 2.4. Antibiotic Resistance Frequencies in C. perfringens

Our online database search revealed that only three studies on the antibiotic resistance of *C. perfringens* strains from Saudi Arabia are available (Table 1 and Table 2). The mean percentages of antibiotic resistance to *C. perfringens* strains were as follows: 21% for metronidazole, 83% for ceftiofur, 39% for clindamycin, 59% for penicillin, 56% for tetracycline, 62% for erythromycin, 47% for oxytetracycline, and 47% for lincomycin. Table 2 shows that the average resistance of *C. perfringens* isolates to metronidazole, ceftiofur, clindamycin, and penicillin increased from 1988 to 2020, although the data are limited.

### 2.5. Meta-Analysis Results

Table 3 summarizes the pooled proportion of resistance of *Clostridium* species for each antibiotic examined. The pooled proportion of resistance was 0.37 (95% CI: 0.22–0.54) and 0.34 (95% CI: 0.23–0.47) for metronidazole and clindamycin, respectively. Similarly, the pooled proportion for tetracycline was 0.34 (95% CI: 0.20–0.51), and a relatively higher resistance was observed for penicillin, with a pooled proportion of 0.45 (95% CI: 0.15–0.78). Although the number of studies involved was small (two studies each), the pooled proportion of resistance for ciprofloxacin, levofloxacin, and erythromycin was 0.30 (95% CI: 0.12–0.57), 0.50 (95% CI: 0.00–1.00), and 0.61 (95% CI: 0.25–0.88), respectively.

**Figure 2:**
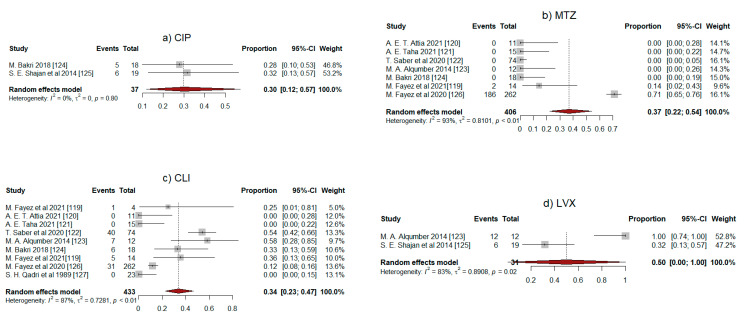

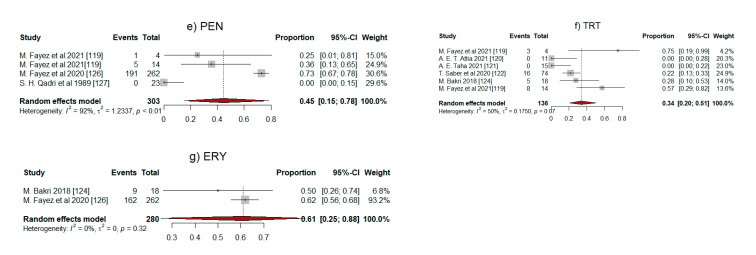
Forest plots of the random-effects meta-analysis of the ten works included in this study. (**a**) CIP, (**b**) MTZ, (**c**) CLI, (**d**) LVX, (**e**) PEN, (**f**) TRT, (**g**) ERY.

Forest plots for each antibiotic are shown in Figure 2a–g. In subgroup analyses, *C. difficile* showed higher resistance to clindamycin (0.41; 95% CI: 0.29–0.55) than *C. perfringens* (0.24; 95% CI: 0.04–0.70), although the difference was not statistically significant (Table 3). Interestingly, for metronidazole, resistance was observed only in *C. perfringens* but not in *C. difficile* (Figure 2b and Table 2). Based on funnel plots of study size against log odds, there was no major publication bias. However, due to the small number of studies, Egger’s test of symmetry was not sufficiently powered to determine publication bias. As there is a contradiction between these two last results, it is not possible to draw any conclusion on this matter.

## 3. Discussion

The information on the antimicrobial susceptibility of the two abovementioned *Clostridium* species, *C. difficile* and *C. perfringens,* has been summarized in this review article. In Saudi Arabia, data on the antimicrobial resistance of *Clostridium* species are extremely limited. The widespread use of antibiotics in the world, as a course plan treatment for different *Clostridium* species, such as *C. difficile* and *C. perfringens,* has led to the development of a new set of *Clostridium* species that are resistant to a variety of antibiotic drugs [7].

Although the data showed that all the *Clostridium* species isolates were still sensitive to metronidazole and vancomycin in Saudi Arabia, it was also noticed that *C. difficile* had become resistant to other antibiotics, including aminoglycosides, lincomycin, clindamycin, and cephalosporins with expanded and extended spectrums, as well as to fluoroquinolones, ampicillin, and amoxicillin, which are routinely used in clinical settings to treat bacterial infections and may increase the risk of CDI [119,120,121].

In this study, we found information about different *C. difficile* isolates from clinical and non-clinical sources (such as baskets, trolleys, conveyor belts, and plastic bags), animals (chicken, cow, sheep, and goat meat), and humans studied in Saudi Arabia and analyzed such information. The resistance rates to levofloxacin, nalidixic acid, ciprofloxacin, and moxifloxacin (a member of the quinolone group) in Saudi Arabia were as high as 100%, 77%, 42%, and 28%, respectively, in *C. difficile* isolates. Moxifloxacin’s total rate of antibiotic resistance in *C. difficile* isolates from clinical and non-clinical specimens in Saudi Arabia was lower compared to that of isolates from European countries such as Poland, the Czech Republic, Germany, Hungary, and France, which showed resistances of 100%, 100%, 68%, 41%, and 38%, respectively [7]. In comparison, the rate of moxifloxacin resistance in clinical isolates from Saudi Arabia was higher than that of isolates from Sweden and Spain, which were 15% and 8%, respectively [7]. Moreover, the moxifloxacin resistance rate in *C. difficile* isolates from Saudi Arabia was lower than that in isolates from North America (reaching 83%) and the United States (reaching 36%), while it was higher than that in isolates from South American countries such as Brazil (which was as low as 8%) [7]. In several countries in the Middle East, close to Saudi Arabia, the moxifloxacin resistance rate in *C. difficile* isolates was lower than that in isolates from Iran (67.9%), South Korea (62.6%), and China (61.8%) but higher than those in strains from Israel (5%) and Japan (0%) [7,122]. From these results, it can be concluded that the resistance rate to quinolones was high in Saudi Arabia, suggesting that caution should be exercised while identifying and treating *C. difficile* infections; we recommend the use of ribotyping methods to determine the drug that should be prescribed.

Clindamycin and erythromycin are lincosamide and macrolide antibiotics, respectively, that are being used in geographical areas with bacteria with significantly high resistance rates [123]. Clindamycin resistance was discovered in 28% of all *C. difficile* isolates from clinical and non-clinical specimens in Saudi Arabia, according to the current investigation. Different reports of clindamycin resistance rates of isolates from different countries were as follows: Clindamycin resistance rates of Saudi Arabian isolates were lower than those of isolates from European countries such as Spain (74%), Sweden (65%), France (34.8%), Poland (38%), and Hungary (31%) [7] and were also lower than those of isolates from the USA (36%), New Zealand (61%), and other Asian countries, such as Iran (84.3%), Japan (87.7%), Korea (81%), and China (88.1%) [7,122]. However, the resistance rate was higher in isolates from the Czech Republic by 10% [7]. Therefore, clindamycin use in Saudi Arabia should be limited to avoid the establishment of resistant strains under selection pressure.

In Saudi Arabia, 50% of all *C. difficile* isolates from clinical and non-clinical specimens tested positive for erythromycin resistance. Recent studies of erythromycin resistance in different countries showed that Saudi Arabian isolate resistance rates were lower than those of European isolates from Poland (100%), the Czech Republic (100%), and Germany (76%) (Banawas, 2018) but higher than those of isolates from Sweden (14%), France (19%), Spain (49%), and Hungary (31%). In addition, North American isolates showed an approximately 86% higher erythromycin resistance rate than Saudi Arabian isolates. Asian countries showed isolates with higher rates of erythromycin resistance compared to that of Saudi Arabian isolates, for example, those isolates from Japan (~88%), Iran (78%), Korea (80%), and China (85%) [7,122]. In our study, the prevalence of tetracycline resistance among *C. difficile* isolates was 24% in Saudi Arabia. This rate was low compared to that of strains from Asian countries such as China (about 63%) and Iran (~33%), but higher than that observed in isolates from countries such as Sweden (~8%), Hungary (12%), and the United States (~8%) [7,122].

*C. perfringens* isolates from Saudi Arabia originated in humans and animals. A high rate of resistance to ceftiofur was observed in isolates from animals. Ceftiofur is a third-generation cephalosporin that is commonly and legally used in veterinary medicine. *C. perfringens* isolates in Saudi Arabia were resistant to ceftiofur (~83%), a high rate compared with that observed in isolates from Brazil (0%), China (2.6%), and Thailand (82%) [124,125,126]. In our study, the erythromycin resistance rate of 62% was higher than that observed in Chinese (49%), Iranian (32.9%), and Brazilian (58.6%) strains [122,125,127]. In addition, the clindamycin resistance rate of 6% seen in Saudi Arabian isolates was lower than that reported in a study on strains from China (26.9%) and higher than that reported for isolates from New Zealand (0%) [125,128]. The strain oxytetracycline resistance rate of 47% seen in Saudi Arabia was lower than that reported for Egypt (67%) and Brazil (48.3%) [93,127]. The metronidazole resistance rate of 21% of strains from Saudi Arabia was higher than that in isolates from Brazil (0%) and New Zealand (0%) [127,128]. Several studies on the prevalence of penicillin-resistant *C. perfringens* strains have been published. In Canada, New Zealand, and Brazil, the rate of penicillin-resistant strains was approximately 0%, which was much lower than that found in Saudi Arabia where the penicillin resistance rate was as high as 59% [127,128,129].

The generalizations of the outcome need to be made in light of the limitations of the study. Some of the important limitations were (i) the small number of total articles published in the area, (ii) the non-performance of quality assessment of the included papers, and (iii) for some of the antibiotics, the number of available articles was less.

## 4. Materials and Methods

### 4.1. Literature Search

An online search was conducted in five databases: ScienceDirect (https://www.sciencedirect.com, accessed on 8 July 2022), PubMed (https://pubmed.ncbi.nlm.nih.gov, accessed on 8 July 2022), Google Scholar (https://scholar.google.com, accessed on 8 July 2022), Web of Science (https://www-webofscience-com, accessed on 8 July 2022), and the Saudi Digital Library (SDL; https://sdl.edu.sa, accessed on 8 July 2022). The search included terms such as “Antibiotic resistance”, “*Clostridium*”, “*C. difficile*”, “*C. botulinum*”, “*C. tetani*”, “*C. perfringens*”, and “Saudi Arabia.” The search was limited to articles published up to December 2021 (Figure 1 and Table 1) either in English or Arabic. In total, 13,535 literature studies were found. Most of the studies, 13,343, with their date studies were excluded either because they were irrelevant or not eligible or had been conducted in other countries.

### 4.2. Selection Criteria

As previously stated, we searched for published studies on the antimicrobial resistance of *Clostridium* species isolated in Saudi Arabia. Except for non-original or duplicate papers, there were no selection restrictions regarding the types of specimens used in the studies (human or animal sources) or the type of investigation followed (Table 1). To reduce selection bias, all potentially relevant publications were rigorously assessed, and only those that met the inclusion criteria were chosen. If a study appeared to be relevant, the complete publications were retrieved after reviewing the abstract (Figure 1).

### 4.3. Data Extraction

Relevant data, such as the location of the study, year of publication, specimen type (animal or human), number of isolates, type of tests used for antimicrobial susceptibility determination, and different resistant strains of *Clostridium* species, were extracted from the selected articles.

### 4.4. Statistical Analysis

To estimate the pooled proportion of resistance to different antibiotics, we employed the *metaprop* command of the “meta” package of R. Due to heterogeneity, we analyzed the results based on a random-effects meta-analysis. We used the inverse variance method for those proportions near boundaries (in this instance, 100% at 1-year survival or 0% at stage IV), which allows computation of exact binomial and score test-based CIs. Whenever the number of studies allowed it, subgroup analysis was performed for *C. difficile* and *C. perfringens* separately, e.g., for clindamycin resistance. Since conventional funnel plots are not appropriate for a low proportion of outcomes, we used funnel plots of study size against log odds to assess publication bias. The frequency of resistance of the isolates was obtained by dividing the number of resistant strains by that of the total isolates (Table 2).

## 5. Conclusions

To the best of our knowledge, this study is the first to evaluate the frequency of antibiotic-resistant *Clostridium* species in Saudi Arabia using a systematic and meta-analysis approach. Our study concluded that, in the case of vancomycin and metronidazole, which are regularly part of the first-line therapy, the rate of appearance of antibiotic resistance was very low among clinical and non-clinical isolates of *C. difficile* in Saudi Arabia. Moreover, other *C. difficile* isolates showed lower resistance rates to drugs, including tetracycline, erythromycin, and ciprofloxacin. Furthermore, clinical *C. perfringens* isolates in Saudi Arabia showed significantly lower resistance rates to vancomycin, metronidazole, and clindamycin. However, resistance rates to ceftiofur, erythromycin, clindamycin, oxytetracycline, metronidazole, and penicillin were high in *C. perfringens* isolates. Therefore, these antibiotics are not recommended in Saudi Arabia for the treatment of clostridial illnesses in humans and animals.

## Figures and Tables

**Table 1:** Search terms used in the electronic database search.

Inclusion Criterium	Search Terms
Antibiotic resistance	Drug resistance, Antimicrobial resistance
*Clostridium* spp.	*C. difficile*, *C. botulinum*, *C. tetani*, *C. perfringens*
Saudi Arabia	Kingdom of Saudi Arabia, SA, KSA, Saudi Arabia

**Table 2:** Antibiotic Susceptibility of *Clostridium* spp. isolated in Saudi Arabia *.

Province/ City	Year	Sample Type (Origin)	Strain (n)	*Clostridium* spp.	AST	Antibiotic Resistance (%)														
						CIP	MTZ	CEF	GEN	CLI	LVX	PEN	VAN	TRT	ERY	OXY	AMP	MXF	LIN	Reference
Al-Ahsa	2019–2020	Camels	4	*C. difficile*	Broth dilution	ND	ND	ND	ND	25	ND	25	ND	75	ND	ND	ND	25	ND	[110]
Al-Jouf	2019	Chickens	11	*C. difficile*	E-tests	ND	0	ND	ND	0	ND	ND	0	0	ND	ND	ND	18	ND	[111]
Al-Jouf	2019	Camels, cows, sheep, and goats	15	*C. difficile*	E-tests	ND	0	ND	ND	0	ND	ND	0	0	ND	ND	ND	20	ND	[112]
Al-Taif	2019	Stools	74	*C. difficile*	E-tests	ND	0	ND	ND	54	ND	ND	0	21.6	ND	ND	ND	48.6	ND	[113]
Al-Bahah and Al-Taif	2011	Baskets, trolleys, conveyor belts, and plastic bags	12	*C. difficile*	E-tests	ND	0	ND	ND	58	100	ND	0	ND	ND	ND	ND	ND	ND	[114]
Jazan	2015	Cow, sheep, and goat meat	18	*C. difficile*	Disk diffusion	27	0	ND	83	33	ND	ND	0	28	50	ND	72	ND	ND	[115]
Eastern province	2011–2012	Stools	19	*C. difficile*	E-tests	30	ND	ND	ND	ND	30	ND	ND	ND	ND	ND	ND	30	ND	[116]
Al-Ahsa	2019–2020	Camels	14	*C. perfringens*	Broth dilution	ND	14	ND	ND	35	ND	35	ND	56	ND	ND	ND	ND	ND	[110]
Eastern province	2018	Dromedary camels, pastures, and herders	262	*C. perfringens*	Broth dilution	ND	27	83	ND	12	ND	73	ND	ND	62	47	ND	ND	47	[117]
Riyadh	1988	Wounds, blood, body fluids, and female genitalia	23	*C. perfringens*	Disk elution	ND	ND	ND	ND	0	ND	0	0	ND	ND	ND	ND	ND	ND	[118]

* AMP, ampicillin; AST, antimicrobial susceptibility test; CEF, ceftiofur; CIP, ciprofloxacin; CLI, clindamycin; ERY, erythromycin; GEN, gentamicin; LIN, lincomycin; LVX, levofloxacin; MTZ, metronidazole; MXF, moxifloxacin; ND, not determined; OXY, oxytetracycline; PEN, penicillin; TRT, tetracycline; VAN, vancomycin.

**Table 3:** Pooled proportion of resistance for *Clostridium* species.

**Antibiotic**	**No. of Studies**	**Pooled Proportion of Resistance (95% CI)**	I^2^ (%) *	***p***
CIP	2	0.30 (0.12–0.57)	0	0.8
MTZ	7	0.37 (0.22–0.54)	93	<0.01
CLI	9	0.34 (0.23–0.47)	87	<0.01
LVX	2	0.50 (0.00–1.00)	83	0.02
PEN	4	0.45 (0.15–0.78)	92	<0.01
TRT	6	0.34 (0.20–0.51)	50	0.07
ERY	2	0.61 (0.25–0.88)	0	0.32

* I^2^: indicates the level of heterogeneity.

## Data Availability

Not applicable.

## Abbreviations

AAD	antibiotic-associated diarrhea
CDI	*C. difficile* infection
CPE	*C. perfringens* enterotoxin
FP	food poisoning
GI	gastrointestinal
NFB	non-foodborne
